# Regional lymph node invasion in pediatric non-rhabdomyosarcoma soft tissue sarcoma: an international cohort study from the International Soft Tissue Sarcoma Consortium

**DOI:** 10.1016/j.eclinm.2025.103409

**Published:** 2025-08-07

**Authors:** Daniel Orbach, Matthieu Carton, Amadeus T. Heinz, Lise Borgwardt, J Herve Brisse, Carlo Morosi, Erin R. Rudzinski, Akmal Safwat, Monika Sparber-Sauer, Stephanie Terezakis, Andrea Ferrari, Aaron R. Weiss, William H. Meyer, David O. Walterhouse, Timothy B. Lautz

**Affiliations:** a SIREDO Oncology Centre (Care, Innovation and Research for Children, Adolescents and Young Adults with Cancer), Institut Curie, Paris, France; b Université Paris Sciences et Lettres, Paris, France; c Biostatistical Unit, Institut Curie, Paris, France; d Department of Pediatric Hematology and Oncology, University Children's Hospital Tuebingen, Tuebingen, Germany; e Stuttgart Cancer Center, Zentrum für Kinder-, Jugend- und Frauenmedizin (Olgahospital), Pädiatrie 5 (Pädiatrische Onkologie, Hämatologie, Immunologie), Klinikum der Landeshauptstadt Stuttgart, Stuttgart, Germany; f Department of Clinical Physiology and Nuclear Medicine, Copenhagen University Hospital-Rigshospitalet, Copenhagen, Denmark; g Imaging Department, Institut Curie, Paris, France; h Department of Radiology, Istituto Nazionale Tumori, Milan, Italy; i Department of Pathology and Laboratory Medicine, Indiana University, 350 W 11th St, Indianapolis, IN, 46202, USA; j Danish Centre for Particle Therapy, Aarhus University Hospital, Aarhus, Denmark; k Klinikum der Landeshauptstadt Stuttgart gKAöR, Olgahospital, Stuttgart Cancer Center, Zentrum für Kinder-, Jugend- und Frauenmedizin, Pädiatrie 5 (Pädiatrische Onkologie, Hämatologie, Immunologie), Stuttgart, Germany; l Radiation Oncology, University of Minnesota, Minneapolis, MN, USA; m Pediatric Oncology Unit, Fondazione IRCCS Istituto Nazionale Tumori, Milan, Italy; n Department of Oncology and Hemato-oncology, University of Milan, Milan, Italy; o Department of Pediatrics, Maine Health Maine Medical Center, Portland, ME, USA; p Jimmy Everest Section of Pediatric Hematology Oncology, Department of Pediatrics, University of Oklahoma Health Sciences Center, Oklahoma City, USA; q Division of Pediatric Hematology/Oncology/Stem Cell Transplant, Department of Pediatrics, Northwestern University Feinberg School of Medicine, Ann & Robert H. Lurie Children's Hospital of Chicago, Chicago, IL, USA; r Division of Pediatric Surgery, Ann & Robert H Lurie Children's Hospital of Chicago, Chicago, IL, USA

**Keywords:** Paediatric/adolescent oncology, Adult type sarcoma, Lymph node invasion, Instruct consortium

## Abstract

**Background:**

In pediatric non-rhabdomyosarcoma soft tissue sarcoma (NRSTS), the frequency and prognostic impact of regional lymph node involvement (N1) are not clearly defined and may vary according to histological type. We therefore to analyze the rate of N1 at diagnosis, the pattern of nodal relapse, and the prognostic impact of N1 in pediatric patients with NRSTS.

**Methods:**

Data were collected and analyzed through the International Soft Tissue SaRcoma ConsorTium (INSTRuCT). Patients aged 0–21 years with NRSTS prospectively enrolled in European and North American cooperative group trials from October 1, 1990 to October 1, 2018 were included. Descriptive statistics, logistic regressions, and Cox proportional hazards models were used to analyze the data and assess prognostic factors.

**Findings:**

1937 patients were eligible for inclusion. The main histotypes were synovial sarcoma (628 cases; 32%), undifferentiated/unclassified sarcoma (396 cases, 20%) and malignant peripheral nerve sheath tumor (275 cases, 14%). Distant metastases were present in 197 (10.2%) patients. N1 was present in 152 (7.8%) patients. With a median follow-up of 7.2 years (95% CI 7.0–7.4), 615 patients (31.7%) had local relapse or progression, 30 (1.5%) had nodal relapse (including four with initial N1), and 287 (14.8%) developed metastases. In multivariate analysis, node positive (N1) status was associated with high pathologic grade (p = 0.010) and distant metastasis (p < 0.0001), but not with tumor size, invasiveness, and histological subgroups (p = 0.36). For non-metastatic tumors (1740 cases), metastatic-free-survival differed between node negative N0 and N1 patients, but overall survival, event-free-survival and nodal relapse-free-interval did not.

**Interpretation:**

N1 is rare in NRSTS during childhood (<8%) and mainly presents in a subset of histotypes. Regional nodal control at 5 years is adequate. However, N1 in NRSTS is a marker of aggressive disease.

**Funding:**

10.13039/100002002Cancer Research Foundation, Children's Research Foundation, Comer Development Board, KickCancer, 10.13039/501100006282King Baudouin Foundation, 10.13039/100003287Rally Foundation for Childhood Cancer Research, 10.13039/100018762Seattle Children's Foundation from Kat's Crew Guild through the 10.13039/100001845Scleroderma Research Foundation, 10.13039/100006058St. Baldrick's Foundation, The Andrew McDonough B+ Foundation, Maddie’s Promise, Summer’s Way Foundation, Friends of T.J. Foundation, Sebastian Strong, Children’s Oncology Group Foundation, and the Sarah Jane Adicoff Endowment for Research in Rhabdomyosarcoma through the 10.13039/100018762Seattle Children's Foundation.

**Research in context**

**Evidence before this study**

Very few studies have analyzed the frequency and the impact of the regional nodal involvement (N1) in children, adolescents and young adults with non-rhabdomyosarcoma soft tissue sarcomas (NRSTS). These studies focused mainly on dedicated histotypes (rhabdoid tumors, synovial sarcoma) and retrospectively analyzed the general therapy strategy of these disease. However, two large studies were conducted by the Children's Oncology Group in the USA (ARSTS-0332 study; 529 patients <30 years with all stages NRSTS), and the European Paediatric Soft Tissue Sarcoma Study Group (EpSSG NRSTS-05 study; 569 patients <25 years with localized NRSTS), analyzed the overall presentation of young patients with NRSTS and the role of different risk factors.

**Added value of this study**

We, analyzing a large amount of patients (1937 patients) prospectively treated for a NRSTS (localized or metastatic) in international cooperative groups, demonstrated that overall N1 is rare in NRSTS during childhood, and mainly present in patients with distant metastasis, with high pathologic grade tumors and were more frequent in some histotypes. We confirmed that, with current treatment guidelines, the regional nodal control is adequate.

**Implications of all the available evidence**

The list of NRSTS during childhood and adolescence that need a systematic regional lymph node radiologic exploration with or without pathology exploration is therefore established and comprises high-grade and metastatic NRSTS, and some specific histologies, even in the absence of clinical suspicious nodes. For low risk tumors of N1, all suspicious enlarged nodes at imaging should require systematic pathological confirmation, to correctly consider initial tumor staging. The favorable outcome of patients with NRSTS and exclusive N1 indicated that they should be treated with curative intent.

## Introduction

The term non-rhabdomyosarcoma soft tissue sarcomas (NRSTS) describes a mixed group of mesenchymal extraskeletal malignancies (that exclude rhabdomyosarcoma) with clinical behavior varying from relatively benign to highly malignant. The rarity, heterogeneity, and aggressiveness of NRSTS make the management of children and adolescents with these tumors complex and challenging.^1^^,^^2^ Retrospective and prospective pediatric studies have found that regional lymph node involvement (N1) in such tumors occurs rarely with numbers ranging from 1.75% to 7.50%.^3^^,^^4^ In the European paediatric Soft tissue Sarcoma Group (EpSSG) experience, only 3% (18/569) of NRSTS presented with N1. However, the precise rate of regional lymph node (LN) involvement is not clearly defined in pediatric NRSTS. Some histotypes have a higher risk of developing regional LN metastases, including epithelioid and clear cells sarcoma.^5^ In contrast, some histotypes rarely develop N1 disease. Only 1/88 (1.1%) and 5/135 (3.7%) patients with non-metastatic synovial sarcoma in the International Society of Paediatric Oncology Malignant Mesenchymal Tumour Committee (SIOP-MMT) and EpSSG studies had N1, respectively.^6^^,^^7^ Notably, the N1 rate was higher in the Cooperative Weichteilsarkom Studiengruppe (CWS) experience with 9% of 432 patients diagnosed with non-metastatic and metastatic synovial sarcoma.^8^ Understanding the incidence of LN involvement in patients with NRSTS is of importance, as both the adult and pediatric literature suggests an association between LN metastasis and inferior overall survival (OS) across all NRSTS subtypes.^5^^,^^9^, ^10^, ^11^, ^12^ In the EpSSG experience, N1 was associated with unfavorable event free survival (EFS; 52.5% for N1 vs. 74.4% for node negative, N0) and OS (62.0% vs. 84.5%) in univariate but not in multivariate analysis (p = 0.80).^13^ In addition, infiltrated lymph nodes may require additional local therapy, such as radiotherapy and/or regional lymph node resection or dissection. In Children's Oncology Group (COG) studies, N1 tumors are classified as high-risk and these patients receive systemic chemotherapy with primary surgery and regional lymph node irradiation.^3^

The objectives of this study are to analyze the frequency, the prognostic impact and the pattern of nodal relapse in NRSTS patients with N1 at diagnosis in a large series of young patients affected by NRSTS.

## Methods

### Study design and participants

The International Soft Tissue Sarcoma Consortium (INSTRuCT) was established in 2017 to foster international research and collaboration focused on pediatric soft tissue sarcoma. The overarching aim is to promote international cooperation and sharing of large well-annotated clinical data from prospective clinical trials. The membership of INSTRuCT is derived from North American and European cooperative groups (COG, EpSSG, and CWS). The INSTRuCT database includes data from studies conducted these groups and data from prior studies sponsored by the International Society of Paediatric Oncology (SIOP) Malignant Mesenchymal Tumour Committee and Italian Association of Pediatric Hematology and Oncology (AIEOP) Soft Tissue Sarcoma Committee. The data generated from these international groups make this the largest database currently available for these tumors.^14^^,^^15^

Included data were from all these groups and previous studies sponsored by the SIOP Malignant Mesenchymal Tumour Committee and Italian Association of Pediatric Hematology and Oncology (AIEOP) Soft Tissue Sarcoma Committee. Data were collected and analyzed through INSTRuCT and from the following North American and European cooperative group prospective studies: Children's Oncology Group (COG), European paediatric soft tissue sarcoma Study Group (EpSSG), CWS and the International Society of Paediatric Oncology Malignant Mesenchymal Tumour Committee (SIOP-MMT).^14^ A data dictionary was generated for NRSTS to define all data elements and values that could be included to the common dataset. Throughout the process, data elements were mapped to the National Cancer Institute thesaurus to adopt standardized terminology.

In this analysis, all patients (aged 0–21 years) prospectively enrolled at diagnosis in European and North American studies, from October 1, 1990 to October 1, 2018, with a NRSTS (World Health Organization histology classification/3), regardless of initial tumor stage, were included.^16^ Where possible, all tumors were graded at diagnosis according to the French pathologic grading system (FNCLCC, Federation nationale des centres de lutte contre le cancer).^17^ Gx tumors are tumors not gradable according to FNCLCC or not graded.

**Fig. 1:**
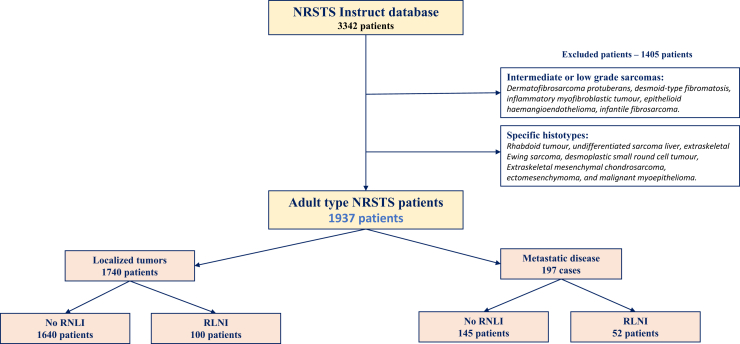
Flow chart of the study.

Some specific histotypes and low grade tumors were excluded, notably tumor types considered benign or intermediate, locally aggressive or intermediate, rarely metastasizing according to the WHO classification system (Fig. 1). Patients were prospectively treated on SIOP-MMT (MMT-95), EpSSG (NRSTS-2005, MTS-2008), CWS (CWS-91, 96, 2002P, IV-2002) and COG (ARST-0332) trials.^3^^,^^5^^,^^6^^,^^18^, ^19^, ^20^, ^21^, ^22^ At diagnosis, recommended initial regional nodal work-up varied according to the period and cooperative groups. Briefly, regional nodal evaluation included clinical examination of all regional nodal areas with or without ultrasound, magnetic resonance imaging (MRI), or [^18^F] fluorodeoxyglucose FDG-PET/CT (-MRI) of the tumor lymph node drainage areas. Fine needle aspiration or biopsy of all suspicious nodes was usually advised. In addition, some groups advised systematic sentinel node exploration and/or regional nodal sampling for some specific histotypes or specific primary tumor locations (e.g., extremity).^4^ In this series, the final nodal status was the one defined by the treating physician, considering overall clinical information, imaging and pathology results. Tumors could have been staged N1, if there was a regional clinically enlarged node cN1 (“lymph node involvement clinically or radiologically assessed” 140 cases) with or without pathology confirmation (pN1, 23 cases). Some tumors were clinically cN0 (“no lymph node involvement clinically or radiologically”) or cNx (“regional lymph nodes cannot be assessed due to lack of information”) but pN1 (12 cases) and were considered N1. Tumors classified as cNx without pathologic or with negative pathologic evaluation (pN0) were considered N0. In all three cooperative groups, lymph node involvement beyond the initial regional basin was considered as metastatic disease. In extremity sarcoma, in-transit nodes, including popliteal/epitrochlear and inguinal/axilla are defined as regional nodes.^4^ Involved lymph nodes beyond the axilla and groin were considered metastatic disease (except primary tumors of the adductor side of the thigh, for which iliac nodes are considered regional).^23^ Overall, distant tumor work up was similar according to the different studies, except for the incorporation of systemic PET-CT in the most recent protocols.

### Ethics

Informed consent from all patients and/or their parents or legal guardians was obtained before inclusion in the initial studies.

The data in the Pediatric Cancer Data Commons (PCDC)—INSTRuCT data commons are de-identified and meet all required regulatory and ethical standards in advance of being ingested into the PCDC.

Name authority granting approval for study: The INternational Soft Tissue saRcoma ConsorTium (INSTRuCT) Executive Committee approved this project.

### Statistical analysis

All data were analyzed using descriptive statistical methods. Associations between N1 and N0 status were assessed using univariable and multivariable logistic regression analyses. For each variable, a likelihood ratio test comparing the model with and without the variable was performed. Odds ratios (ORs) were reported along with their 95% CIs. Survival time was calculated from the date of diagnosis (initial biopsy/surgery) to the time of last follow-up or an event, OS as the time from diagnosis to the date of death from any cause, EFS as the time from diagnosis to the first event (local progression, relapse, metastasis, death) and metastatic-free survival (MFS) as the time from diagnosis to metastatic event or death during follow-up. Nodal relapse free Interval (NRFI) was defined as the time from the date of diagnosis to regional lymph node progression/relapse. Patients alive without recurrence at last contact were censored at the date of last follow-up. Survival curves were plotted according to the Kaplan–Meier method. p values below 0.05 were considered significant. The prognostic role of factors was investigated by comparing Kaplan–Meier curves using log-rank tests and Cox univariate models. The proportional hazards assumption for the Cox models were checked using Schoenfeld residuals. The global test, as well as tests for individual covariates, did not indicate significant violations of the proportional hazards assumption. Multivariate analysis was performed using the Cox proportional-hazards regression model with a backward procedure to identify the variables retained with a p-value below 0.20. In multivariate analysis, small groups defined as less than 40 cases, were analyzed as a common “other histotypes” group. Median follow-up (from age at diagnosis to age of death or age at last follow-up) with its 95% CI was described according to the reverse Kaplan–Meier method.^24^ No imputation for missing value was performed.

### Role of funding source

The International Soft Tissue Sarcoma Consortium and the Pediatric Cancer Data Commons are supported in part by Cancer Research Foundation, Children's Research Foundation, Comer Development Board, KickCancer, King Baudouin Foundation, Rally Foundation for Childhood Cancer Research, Seattle Children's Foundation from Kat's Crew Guild through the Sarcoma Research Fund, St Baldrick's Foundation, The Andrew McDonough B + Foundation, Maddie's Promise, Summer's Way Foundation, Friends of T.J. Foundation, Sebastian Strong, Children's Oncology Group Foundation, and the Sarah Jane Adicoff Endowment for Research in Rhabdomyosarcoma through the Seattle Children's Foundation. This work is made possible through the efforts of Children's Oncology Group, Cooperative Weichteilsarkom Studiengruppe der GPOH (CWS), The European paediatric Soft tissue sarcoma Study Group, MMT Malignant Mesenchymal Tumour Committee, STSC AIEOP Italian Soft Tissue Sarcoma Committee. Funders had no role in study design, data collection, data analyses, interpretation, or writing of report. A private grant (S. Wisnia) paid publication fees.

## Results

Overall, 1937 patients with NRSTS were analyzed (Fig. 1). Patients were treated on studies among EpSSG (570 cases; 29%), CWS (539 cases; 28%), COG (457 cases; 24%) and SIOP-MMT (371 cases; 19%) (Table 1). Median age at diagnosis was 12.8 years (Quartile Q1, Q3: 8.5–15.3). The most frequent primary site was extremity (53%). Tumors were >5 cm in 59% of cases (1039/1766 cases). The most frequent histotypes were synovial sarcoma (biphasic, spindle-cell or not otherwise specified (NOS)) with 628 cases (32%), malignant peripheral nerve sheath tumor (275 cases (14%), undifferentiated/unclassified sarcoma NOS 317 cases (16%), epithelioid sarcoma 123 cases (6.4%) and alveolar soft-part sarcoma 96 cases (5.0%; Table 2). Overall, distant metastases were present in 197 cases (10%). N1 was present in 152 patients (7.8%) with pathologic confirmation (pN1) in only 35/152 cases (23.0%). Among the 1740 patients with non-metastatic tumors, 100 had N1 (5.7%) including 18 pN1 (1.0%).

**Table 1:** Patients and tumor characteristics according to the presence of a regional nodal invasion (N1) or not (N0) at diagnosis.

Characteristic	N0 N = 1785	N1 N = 152	Total N = 1937
**Patient sex**			
Female	862 (48%)	72 (47%)	934 (48%)
Male	921 (52%)	80 (53%)	1001 (52%)
Unknown	2 (0.1%)	0 (0%)	2 (0.1%)
**Age at diagnosis (years)**			
Median (Q1, Q3)	12.8 (8.4, 15.4)	12.5 (8.6, 14.6)	12.8 (8.5, 15.3)
Mean (SD)	11.6 (5.1)	11.1 (5.2)	11.6 (5.1)
**Year of diagnosis**			
1988–1999	446 (25%)	30 (20%)	476 (25%)
2000–2009	788 (44%)	62 (41%)	850 (44%)
2010–2016	551 (31%)	60 (39%)	611 (32%)
**Data contributor**			
COG	398 (22%)	59 (39%)	457 (24%)
CWS	496 (28%)	43 (28%)	539 (28%)
EpSSG	539 (30%)	31 (20%)	570 (29%)
SIOP-MMT	352 (20%)	19 (13%)	371 (19%)
**Study**			
ARST0332	398 (22%)	59 (39%)	457 (24%)
CWS-IV-2002	3 (0.2%)	1 (0.7%)	4 (0.2%)
CWS2002p	190 (11%)	14 (9.2%)	204 (11%)
CWS91	90 (5.0%)	2 (1.3%)	92 (4.7%)
CWS96	213 (12%)	26 (17%)	239 (12%)
MTS2008_NRSTS	19 (1.1%)	9 (5.9%)	28 (1.4%)
NRSTS2005	520 (29%)	22 (14%)	542 (28%)
MMT 95	352 (20%)	19 (13%)	371 (19%)
**Tumor site**			
Extremities	954 (53%)	75 (49%)	1029 (53%)
Trunk	503 (28%)	53 (35%)	556 (29%)
Head and Neck	267 (15%)	21 (14%)	288 (15%)
NA	61 (3.4%)	3 (2.0%)	64 (3.3%)
**Tumor size**			
≤5 cm	694 (43%)	33 (24%)	727 (41%)
>5 cm	934 (57%)	105 (76%)	1039 (59%)
Unknown	157	14	171
**Invasiveness**			
T1 Stage	1016 (57%)	57 (38%)	1073 (55%)
T2 Stage	681 (38%)	90 (59%)	771 (40%)
TX Stage	88 (4.9%)	5 (3.3%)	93 (4.8%)
**IRS group**			
IRS 1	434 (36%)	–	434 (34%)
IRS 2	305 (26%)	17 (18%)	322 (25%)
IRS 3	307 (26%)	24 (26%)	331 (26%)
IRS 4	145 (12%)	52 (56%)	197 (15%)
Unknown	594	59	653

Abbreviations: COG, Children’s Oncology Group; CWS, Cooperative Weichteilsarkom Studiengruppe; EpSSG, European paediatric Soft tissue Sarcoma Group; Max, Maximum; Min, minimum; Q, quartile; MMT, Malignant Mesenchymal Tumour study group; NA, not available; SD, Standard deviation; T1, Tumor confined to tissue of origin; T2, Tumour extending beyond tissue of origin; Tx, Tumor extent unknown.

**Table 2:** Regional node involvement rate according to pathology characteristics.

Tumor characteristics	N1 Rate	p-value
**Histological type**		0.013
**Frequent N1** (>10%)		
Solitary Fibrous tumour, malignant (8815/3)	1/4 (25%)	
Extraskeletal myxoid chondrosarcoma (9231/3)	1/5 (20%)	
Angiosarcoma of Soft Tissue (9120/3)	5/27 (19%)	
Epithelioid sarcoma (8804/3)	18/123 (15%)	
Undifferentiated pleomorphic sarcoma (8802/3)	4/29 (14%)	
Clear cell sarcoma of soft tissue (9044/3)	6/52 (12%)	
Undifferentiated/unclassified sarcoma NOS (8805/3)	35/317 (11%)	
Ectomesenchymoma (8921/3)	1/9 (11%)	
**Possible N1** (5–9%)		
Myxoid Liposarcoma (8852/3)	2/23 (8.7%)	
Malignant peripheral nerve sheath tumor (9540/3)	22/275 (8.0%)	
Low-grade myofibroblastic sarcoma (8825/3)	1/13 (7.7%)	
Synovial sarcoma (NOS, biphasic, SC)	44/628 (7.0%)	
Extraskeletal Mesenchymal Chondrosarcoma (9240/3)	2/35 (5.7%)	
Undifferentiated/unclassified round cell sarcoma (8803/3)	1/19 (5.3%)	
**Rarely N1** (<5%)		
Alveolar soft-part sarcoma (9581/3)	4/96 (4.2%)	
Leiomyosarcoma (Excluding Skin) (8890/3)	2/63 (3.2%)	
Undifferentiated/unclassified spindle cell sarcoma (8801/3)	1/31 (3.2%)	
Liposarcoma, NOS (8850/3)	1/54 (1.9%)	
Adult Fibrosarcoma (8810/3)	1/62 (1.6%)	
Low-Grade Fibromyxoid Sarcoma (8840/3)	0/41 (0.0%)	
Malignant Triton Tumour (9561/3)	0/4 (0.0%)	
Myoepithelioma carcinoma (8982/3)	0/7 (0.0%)	
Myxofibrosarcoma (8811/3)	0/3 (0.0%)	
PEComa NOS, malignant (8714/3)	0/5 (0.0%)	
Pleomorphic Liposarcoma (8854/3)	0/4 (0.0%)	
Sclerosing Epithelioid Fibrosarcoma (8840/3)	0/2 (0.0%)	
Epithelioid Malignant peripheral nerve sheath tumor (9542/3)	0/6 (0.0%)	
**Histologic grade**		<0.0001
FNCLCC, Grade 1	3/140 (2.1%)	
FNCLCC, Grade 2	31/466 (6.7%)	
FNCLCC, Grade 3	57/414 (14%)	
FNCLCC, Grade Gx	61/917 (6.7%)	

Abbreviation: FNCLCC, Fédération Nationale des Centres de Lutte Contre le Cancer (French National Centers Federation against cancer).

For tumor histotypes with more than 10 cases, N1 was mainly observed in patients with angiosarcoma (5/27 cases; 19%), clear cell sarcoma (6/52; 12%), epithelioid sarcoma (18/123; 15%), undifferentiated pleomorphic (4/29; 14%) and undifferentiated/unclassified sarcomas NOS (35/317; 11%). In contrast, N1 was rare (i.e., <5%) in alveolar soft-part sarcoma, leiomyosarcoma, liposarcoma, adult-type fibrosarcoma, undifferentiated/unclassified spindle cell sarcoma and low-grade fibromyxoid sarcoma (Table 2). In univariate analysis, gender, age as a continuous variable, year of diagnosis, or tumor site (extremity/trunk, head and neck) were not associated with N1. The rate of N1 correlated with tumor size, invasiveness (T stage), pathologic grade, presence of distant metastasis and histotype. However, in the multivariate analysis, only pathological grade and presence of metastases significantly correlated with the presence of N1 (Table 3).

**Table 3:** Univariate and multivariate analysis for factors associated to regional nodal invasion.

Characteristic	N1 rate	Univariate			Multivariable		
		OR^a^	95% CI^a^	p-value	OR^a^	95% CI^a^	p-value
**Tumor size**				<0.0001			0.12
≤5 cm	33/727 (4.5%)	1.00	–		–	–	
>5 cm	105/1039 (10%)	2.36	1.60, 3.59		1.44	0.91, 2.30	
**Invasiveness**				<0.0001			0.20
T1 Stage Finding	49/975 (5.0%)	1.00	–		–	–	
T2 Stage Finding	87/742 (12%)	2.51	1.75, 3.63		1.40	0.93, 2.13	
TX Stage Finding	2/49 (4.1%)	0.80	0.13, 2.71		0.58	0.09, 2.07	
**Histology group**				<0.0001			0.30
Synovial sarcoma (NOS, biphasic, SC) (9040/3; 9041/3, 9043/3)	38/594 (6.4%)	1.00	–		–	–	
Adult Fibrosarcoma (8810/3)	1/57 (1.8%)	0.26	0.01, 1.24		0.37	0.02, 1.77	
Alveolar soft-part sarcoma (9581/3)	4/90 (4.4%)	0.68	0.20, 1.75		0.53	0.15, 1.44	
Clear cell sarcoma of soft tissue (9044/3)	6/48 (13%)	2.09	0.76, 4.90		2.22	0.75, 5.69	
Epithelioid sarcoma (8804/3)	16/105 (15%)	2.63	1.37, 4.84		2.06	1.02, 4.00	
Leiomyosarcoma (Excluding Skin) (8890/3)	2/57 (3.5%)	0.53	0.09, 1.80		0.82	0.13, 2.90	
Liposarcoma, NOS (8850/3)	1/52 (1.9%)	0.29	0.02, 1.37		0.58	0.03, 3.00	
Malignant peripheral nerve sheath tumor (9540/3)	21/260 (8.1%)	1.29	0.73, 2.22		1.09	0.60, 1.95	
“Other histotypes”	14/217 (6.5%)	1.01	0.52, 1.86		1.06	0.53, 2.04	
Undifferentiated/unclassified sarcoma NOS (8805/3)	35/286 (12%)	2.04	1.26, 3.31		1.25	0.74, 2.11	
**Histologic grade**				<0.0001			0.010
FNCLCC, Grade 1	2/127 (1.6%)	1.00	–		–	–	
FNCLCC, Grade 2	26/414 (6.3%)	4.19	1.23, 26.2		3.04	0.82, 19.9	
FNCLCC, Grade 3	56/386 (15%)	10.6	3.24, 65.3		5.11	1.43, 32.9	
FNCLCC, Grade G_X_	54/839 (6.4%)	4.30	1.32, 26.5		2.88	0.82, 18.4	
**Metastatic at diagnosis**				<0.0001			<0.0001
No	88/1583 (5.6%)	1.00	–		–	–	
Yes	50/183 (27%)	6.39	4.31, 9.40		4.39	2.86, 6.71	

Abbreviations: FNCLCC, Fédération Nationale des Centres de Lutte Contre le Cancer (French National Centers Federation against cancer); NOS, non-other specified; SC, spindle cells, T1, Tumor confined to tissue of origin; T2, Tumor extending beyond tissue of origin; Tx, Tumor extent unknown.

a OR, Odds Ratio; CI, Confidence Interval.

**Fig. 2:**
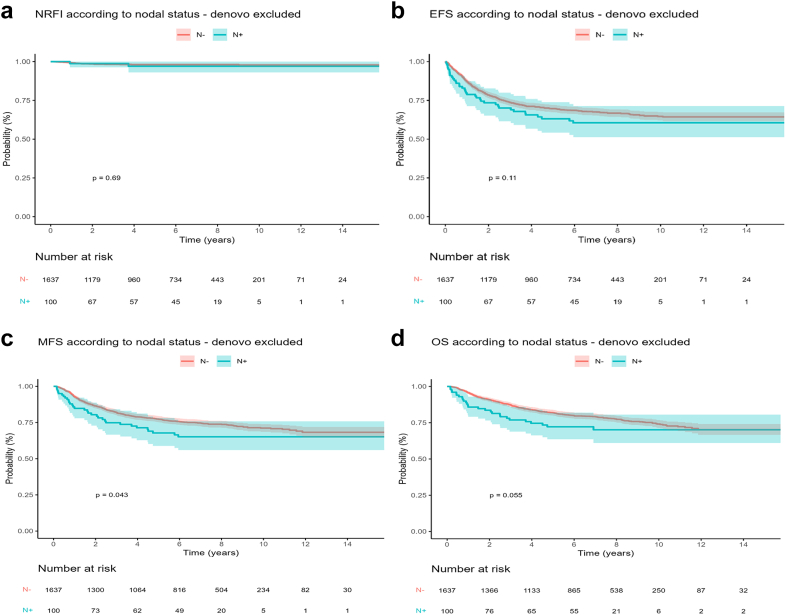
Prognostic impact of initial regional lymph node invasion (N1) in localized non-rhabdomyosarcoma soft tissue sarcoma (NRSTS). Nodal relapse free interval (Fig. 2a), event free survival (Fig. 2b), metastasis free survival (Fig. 2c) and overall survival (Fig. 2d). Red line: no N1 at diagnosis, blue line: presence of N1.

Among the 1740 patients with non-metastatic tumors, N1 was related to tumor size, invasiveness, histotype, and pathologic grade. In multivariate analysis, only pathologic grade remained significant. During follow-up of patients with non-metastatic disease (n = 1740), regional nodal relapse occurred in 28 cases (isolated nodal relapse in 19 cases, 1.6%), including two who suffered from initial N1 (2/152, 1.3%), 404 patients encountered local relapse or progression (23.2%) and 207 patients (11.9%) developed distant metastases, with some patients having multiple sites of relapse. No tumor event occurred in 1242 patients (71.4%). MFS differed between N0 vs. N1 patients but OS, EFS, and NRFI did not: 5 years MFS 77.3% (95% CI 75.2–79.5) vs. 67.8% (58.9–78.1; p = 0.043); OS 81.9% (79.9–83.9) vs. 72.2% (63.6–81.9; p = 0.055); EFS 69.6% (67.3–72.0) vs. 63.2% (54.1–73.8; p = 0.11), and NRFI was 98.1% (97.4–98.8) vs. 97.0% (93.0–100.0; p = 0.69; Fig. 2), respectively. Unfavorable risk factors associated with EFS and OS were tumor size, site, invasiveness, histologic and pathologic grade, but not nodal status at diagnosis or period of recruitment (Supplemental Tables S1 and S2).

Details of delivered therapy for the entire population are provided in Table 4. For all patients (n = 1937), including those with metastatic disease at diagnosis, with a median follow-up of 7.2 years, 30 patients had a nodal relapse (isolated in 19 cases, 0.9%), including four after initial N1 (n = 4/152, 2.6%), 615 had local primary relapse or progression (31.7%) and 287 (14.8%) developed distant metastases, including 80 initially with metastases. Notably, multiple sites of tumor events could be possible. Overall, 1322 patients had no tumor event recorded (68.2%). At the time of last follow-up, 1435 patients (74%) were alive (76% for N0 and 58% for N1). The main cause of death was disease progression (450/499 cases, 90%). For the entire cohort (1937 cases), for N0 vs. N1 patients, the 5-years MFS was 73.0% (70.9–75.2) vs. 54.2% (46.5–63.2; p < 0.0001), OS 77.9% (75.9–80.0) vs. 57.6% (49.8–66.5; p < 0.0001), EFS 65.7% (63.5–68.1) vs. 50.7% (43.0–59.7; p < 0.0001) and NRFI is 98.2% (97.5–98.9) vs. 95.9% (91.9–100.0; p = 0.16, Supplemental Figure S1), respectively.

**Table 4:** Therapy delivered according to the presence of a N1 (N1) or not (N0) at diagnosis.

Characteristic	Number of patients analyzed	Nodal status		Overall N = 1937^a^
		N0 N = 1,785^a^	N1 N = 152^a^	
Chemotherapy	1908	1067 (61%)	114 (77%)	1181 (62%)
Unknown		25	4	29
Radiotherapy at primary	1893	782 (45%)	79 (54%)	861 (45%)
Unknown		36	5	41
Dose at primary (Gray)	790			
Median (Q1, Q3)		50.4 (45.0, 54.0)	52.6 (48.6, 55.8)	50.4 (45.0, 54.0)
Mean (SD)		50.1 (7.1)	51.8 (6.5)	50.7 (7.1)
Radiotherapy at nodes area	1889	15 (0.9%)	24 (17%)	39 (2.1%)
Unknown		40	8	48
Dose at nodes (Gray)	31			
Median (Q1, Q3)		45.0 (41.4, 54.0)	45.0 (45.0, 50.3)	45.0 (44.8, 50.4)
Mean (SD)		46.9 (7.6)	47.7 (4.9)	47.4 (5.9)

Abbreviations: Q, Quartile; SD, Standard deviation.

a Number (%).

## Discussion

We report the rate of N1 at diagnosis, the pattern of nodal relapse, and the prognostic impact of N1 in a large series of pediatric patients with NRSTS by analyzing prospective data. The data generated from these international groups make this the largest database currently available for these tumors. Overall, N1 is rare and affects less than 10% of patients, in agreement with the Surveillance, Epidemiology, and End Results experience.^11^ For the entire cohort, N1 was associated with high pathologic grade and/or metastatic disease. With this analysis, we can demonstrate that not only angiosarcoma, clear cell sarcoma and epithelioid sarcoma,^5^^,^^25^ but also undifferentiated pleomorphic and undifferentiated/unclassified sarcomas NOS were correlated with a high risk of N1. Greater than 10% of patients with these histotypes had N1. In these high-risk sarcomas, comprehensive imaging of the regional LN basin(s) with CT or MRI as well as PET-CT is essential and should be considered to systematically look for evidence of nodal involvement. Given the implications for treatment and prognosis, radiographically abnormal lymph nodes should be sampled to confirm the presence of tumor. More controversial is the question of regional lymph node sampling, preferably with a sentinel lymph node technique, for patients with these high-risk tumors who have no evidence of lymph node involvement on imaging.^4^ While sentinel lymph node biopsy can identify tumor involvement in radiographically and clinically normal lymph nodes,^26^ the oncologic benefit of diagnosing this micro-metastatic disease is unknown. In contrast, some tumors are at low risk (<5% of patients N1): alveolar soft-part sarcoma, leiomyosarcoma, liposarcoma, adult-type fibrosarcoma, undifferentiated/unclassified spindle cell sarcoma and low-grade fibromyxoid sarcoma. In these histotypes, N0 at imaging should not lead to systematic tumor sampling but suspicious N1 at imaging would require systematic pathologic confirmation.

The adult community edited, in the framework of ESMO (European Society of Medical Oncology), EURACAN (European Reference Network for Rare Adult Solid Cancers) and GENTURIS (European Reference Network for Genetic Tumour Risk Syndromes) societies, clinical practice guidelines for diagnosis, management and follow-up of soft tissue sarcomas.^27^ They confirm that regional lymph node (LN) metastases are also rare (i.e., < 1%) in adults with soft tissue sarcomas with the exceptions of epithelioid sarcoma, clear-cell sarcoma, synovial sarcoma and angiosarcomas, for which regional assessment by CT/MRI may be added to the usual staging procedures. They confirm that regional LN metastases constitute an adverse prognostic factor. They indicate that regional LN metastases should be distinguished from soft tissue metastases in a LN basin. In angiosarcoma, a targeted dissection should be done in patients with clinically- and/or radiologically-concerning nodes, as the LN metastatic risk is higher than other NRSTS histotypes (5%–15%). FDG-PET/CT may be reserved as an adjunct, for example for characterizing equivocal CT LN findings in relevant sarcoma types. Even though N1 patients have unfavorable outcomes, with worse OS and MFS than N0 patients, this factor is not an independent risk factor in multivariate analysis and N-stage therefore represents a marker of aggressive disease. In the Surveillance, Epidemiology, and End Results database, among 941 patients with NRSTS, the presence of a N1 was also associated with unfavorable OS and cause specific survival (p < 0.04) in multivariate analysis.^11^ In addition, the narrow gap between 5-year EFS and OS, in the total population and in those with non-metastatic disease, illustrates the extremely poor prognosis for patients with relapsed NRSTS irrespective of the site of relapse, as described for rhabdomyosarcoma with N1.^28^

N1 seems more frequent in recent NRSTS studies, with 9.8% after 2010 (60/611 cases) vs. 6.3% before 2000 (30/476). It seems that these results should be interpreted as a change in practice over time, with more radiological evaluation in the most recent protocols than differences in biological behavior. Notably, in the COG ARST0332 study, the overall rate of patients with N1 tumors was 3.7% (20/529). Among these patients, 19/20 had enlarged lymph nodes on pretreatment imaging. What can be emphasized is that N1 alone was no major adverse prognostic factor in pediatric NRSTS compared to metastatic disease, as the 5-years OS was 85.7% for patients with N1 disease only compared to 15.4% in patients with N1 and distant metastases. Of the entire cohort, only three patients had lymph nodes metastasis at first recurrence, two of whom had negative lymph node biopsies at study entry. The authors suggested that systematic LN sampling might not be necessary for young patients without clinically or radiologically evident lymphadenopathy with the possible exception of >5 cm epithelioid sarcoma and clear cell sarcoma where the incidence of nodal involvement is much higher.^5^

The authors acknowledge some limitations of this study. These include the definition of N1 at diagnosis was not consistent among the cooperative groups and the initial nodal evaluation varied by cooperative group, specific study, and period of treatment. In addition, some data are missing on the detailed treatment of regional nodal extension. Finally, some of the delayed regional nodal biopsy or dissection data and pathologic grade data were missing, due to the long period of analysis and retrospective character of the study.

This large study confirms that overall regional node tumor control seems adequate with a 5-year lymph node control rate of 95.9% (95% CI, 91.9–100%) showing that nodal relapse is rare in pediatric NRSTS given current treatment strategies. Furthermore, the grading and initial tumor staging of the NRSTS appear here essential. The authors propose that at diagnosis, lymph node biopsy should systematically be performed in patients with NRSTS and clinically positive, radiologically positive, or suspicious nodes (cN1). In addition, systematic radiographic imaging of regional lymph nodes should be considered in patients with high-grade NRSTS or specific histologies, including clear cell sarcoma, epithelioid sarcoma, angiosarcoma, undifferentiated pleomorphic and undifferentiated/unclassified NOS sarcomas. To harmonize the definition on nodal extension supporting further investigation of this topic, an international consensus has been recently proposed in children with soft tissue sarcomas.^4^

## Contributors

All authors read and approved the final version of the manuscript.

**Orbach Daniel** (Author that directly accessed and verified the data reported in the manuscript) conceptualisation, data curation, formal analysis, investigation, methodology, resources, validation, visualisation, writing—original draft, and writing—review & editing.

**Carton Matthieu** (Author that directly accessed and verified the data reported in the manuscript) conceptualisation, data curation, formal analysis, methodology, validation, visualisation, writing original draft, and writing review & editing.

**Heinz Amadeus T** data curation, formal analysis, methodology, project administration, supervision, validation, visualisation, writing—original draft, and writing review & editing.

**Borgwardt Lise** writing review & editing.

**Brisse J Herve** writing review & editing.

**Morosi Carlo** writing review & editing.

**Rudzinski Erin R** writing review & editing.

**Safwat Akmal** writing review & editing.

**Sparber-Sauer Monika** writing review & editing.

**Terezakis Stephanie** writing review & editing.

**Ferrari Andrea** conceptualisation, data curation, formal analysis, methodology, project administration, supervision, validation, visualisation, writing—original draft, and writing review & editing.

**Weiss Aaron R** conceptualisation, data curation, formal analysis, methodology, project administration, supervision, validation, visualisation, writing—original draft, and writing review & editing.

**Meyer William H** writing review & editing.

**Walterhouse David O** writing review & editing.

**Lautz Timothy B** conceptualisation, data curation, formal analysis, methodology, project administration, supervision, validation, visualisation, writing—original draft, and writing review & editing.

## Data sharing statement

Data available upon request, data dictionary defining each fields in the set, statistical analysis detailed, overall deidentified participant results.

## Declaration of interests

The authors declare that they have no known competing financial interests or personal relationships that could have appeared to influence the work reported in this paper.
