# Supplementary material for: Regional lymph node invasion in pediatric non-rhabdomyosarcoma soft tissue sarcoma: an international cohort study from the International Soft Tissue Sarcoma Consortium
Source: eClinicalMedicine. 2025 Aug 7;87:103409. doi: 10.1016/j.eclinm.2025.103409 (PMC12355411; doi:10.1016/j.eclinm.2025.103409)
Supplement: Supplemental Figure and Tables [file mmc1.docx]

|  | | | Univariate Cox regression | | Multivariate Cox regression | |
| --- | --- | --- | --- | --- | --- | --- |
|  | **N** | **Event N** | **HR [95% CI]** | **p-value** | **HR [95% CI]** | **p-value** |
| **Nodal status at diagnosis** |  |  |  | 0.30 |  | 0.94 |
| N- | 1,453 | 464 | — |  | — |  |
| N+ | 83 | 31 | 1.25 [0.87 - 1.79] |  | 0.99 [0.68 - 1.43] |  |
| **Year of diagnosis** |  |  |  | 0.08 |  | 0.39 |
| 1988-1999 | 364 | 135 | — |  | — |  |
| 2000-2009 | 685 | 216 | 0.79 [0.64 - 0.99] |  | 0.86 [0.68 - 1.08] |  |
| 2010-2016 | 487 | 144 | 0.79 [0.62 - 1.00] |  | 0.85 [0.64 - 1.12] |  |
| **Tumor size** |  |  |  | **<0.0001** |  | **<0.0001** |
| <=5 cm | 677 | 152 | — |  | — |  |
| >5 cm | 859 | 343 | 2.06 [1.70 - 2.50] |  | 1.76 [1.41 - 2.20] |  |
| **Invasiveness** |  |  |  | **<0.0001** |  | **<0.0001** |
| T1 Stage Finding | 902 | 210 | — |  | — |  |
| T2 Stage Finding | 597 | 275 | 2.35 [1.96 - 2.81] |  | 1.65 [1.34 - 2.02] |  |
| TX Stage Finding | 37 | 10 | 1.29 [0.68 - 2.44] |  | 1.27 [0.67 - 2.42] |  |
| **Tumor site** |  |  |  | **<0.0001** |  | **0.0002** |
| Limbs | 835 | 213 | — |  | — |  |
| Trunk | 431 | 185 | 1.96 [1.61 - 2.39] |  | 1.50 [1.20 - 1.89] |  |
| Head and Neck | 239 | 86 | 1.67 [1.30 - 2.15] |  | 1.67 [1.28 - 2.19] |  |
| NA | 31 | 11 | 1.68 [0.92 - 3.09] |  | 1.52 [0.82 - 2.83] |  |
| **Histology** |  |  |  | **<0.0001** |  | **<0.0001** |
| Synovial sarcoma (NOS, biphasic, SC) (9040/3; 9041/3, 9043/3) | 549 | 147 | — |  | — |  |
| Adult Fibrosarcoma (8810/3) | 53 | 9 | 0.77 [0.39 - 1.50] |  | 0.65 [0.33 - 1.29] |  |
| Alveolar soft-part sarcoma (9581/3) | 68 | 19 | 0.93 [0.58 - 1.50] |  | 1.32 [0.80 - 2.15] |  |
| Clear cell sarcoma of soft tissue (9044/3) | 39 | 20 | 2.14 [1.34 - 3.41] |  | 3.42 [2.10 - 5.55] |  |
| Epithelioid sarcoma (8804/3) | 85 | 34 | 1.84 [1.26 - 2.67] |  | 2.07 [1.41 - 3.04] |  |
| Leiomyosarcoma (Excluding Skin) (8890/3) | 48 | 5 | 0.37 [0.15 - 0.91] |  | 0.43 [0.18 - 1.07] |  |
| Liposarcoma, NOS (8850/3) | 50 | 9 | 0.69 [0.35 - 1.35] |  | 1.15 [0.57 - 2.35] |  |
| Malignant peripheral nerve sheath tumor (9540/3) | 229 | 111 | 2.39 [1.86 - 3.05] |  | 1.89 [1.46 - 2.46] |  |
| Other | 188 | 56 | 1.26 [0.92 - 1.71] |  | 1.22 [0.88 - 1.68] |  |
| Undifferentiated sarcoma NOS (8805/3) | 227 | 85 | 1.62 [1.24 - 2.11] |  | 1.03 [0.76 - 1.40] |  |
| **Histological grade (FNCLCC)** |  |  |  | **<0.0001** |  | **0.0021** |
| Grade 1 | 122 | 14 | — |  | — |  |
| Grade 2 | 375 | 109 | 2.64 [1.51 - 4.61] |  | 2.62 [1.45 - 4.71] |  |
| Grade 3 | 307 | 117 | 3.82 [2.19 - 6.65] |  | 2.45 [1.36 - 4.43] |  |
| Grade GX | 732 | 255 | 3.42 [2.00 - 5.85] |  | 2.70 [1.51 - 4.81] |  |

**Abbreviations:** *FNCLCC, Fédération Nationale des Centres de Lutte Contre le Cancer (French National Centers Federation against cancer); NA, not available; NOS, non-other specified; SC, spindle cells, T1, Tumor confined to tissue of origin; T2, Tumor extending beyond tissue of origin; Tx, Tumor extent unknown.*

**Supplemental Table 1.** Risk factors associated to event free survival for localized tumors (univariate and multivariate analyzes).

|  | | | Univariate Cox regression | | Multivariate Cox regression | |
| --- | --- | --- | --- | --- | --- | --- |
|  | **N** | **Event N** | **HR [95% CI]** | **p-value** | **HR [95% CI]** | **p-value** |
| **Nodal status at diagnosis** |  |  |  | 0.26 |  | 0.99 |
| N- | 1,453 | 317 | — |  | — |  |
| N+ | 83 | 21 | 1.30 [0.84 - 2.03] |  | 1.00 [0.64 - 1.56] |  |
| **Year of diagnosis** |  |  |  | **0.036** |  | 0.21 |
| 1988-1999 | 364 | 102 | — |  | — |  |
| 2000-2009 | 685 | 149 | 0.74 [0.57 - 0.95] |  | 0.82 [0.62 - 1.07] |  |
| 2010-2016 | 487 | 87 | 0.72 [0.54 - 0.96] |  | 0.75 [0.54 - 1.05] |  |
| **Tumor size** |  |  |  | **<0.0001** |  | **<0.0001** |
| <=5 cm | 677 | 76 | — |  | — |  |
| >5 cm | 859 | 262 | 3.16 [2.44 - 4.07] |  | 2.57 [1.92 - 3.45] |  |
| **Invasiveness** |  |  |  | **<0.0001** |  | **<0.0001** |
| T1 Stage Finding | 902 | 117 | — |  | — |  |
| T2 Stage Finding | 597 | 214 | 3.19 [2.54 - 3.99] |  | 1.84 [1.43 - 2.37] |  |
| TX Stage Finding | 37 | 7 | 1.63 [0.76 - 3.49] |  | 1.47 [0.68 - 3.17] |  |
| **Tumor site** |  |  |  | **<0.0001** |  | **<0.0001** |
| Limbs | 835 | 125 | — |  | — |  |
| Trunk | 431 | 142 | 2.54 [2.00 - 3.24] |  | 1.74 [1.32 - 2.29] |  |
| Head and Neck | 239 | 62 | 2.02 [1.49 - 2.73] |  | 2.07 [1.49 - 2.87] |  |
| NA | 31 | 9 | 2.34 [1.19 - 4.61] |  | 2.06 [1.03 - 4.13] |  |
| **Histology** |  |  |  | **<0.0001** |  | **<0.0001** |
| Synovial sarcoma (NOS, biphasic, SC) (9040/3; 9041/3, 9043/3) | 549 | 93 | — |  | — |  |
| Adult Fibrosarcoma (8810/3) | 53 | 5 | 0.69 [0.28 - 1.70] |  | 0.56 [0.23 - 1.39] |  |
| Alveolar soft-part sarcoma (9581/3) | 68 | 5 | 0.39 [0.16 - 0.95] |  | 0.68 [0.27 - 1.70] |  |
| Clear cell sarcoma of soft tissue (9044/3) | 39 | 15 | 2.51 [1.45 - 4.32] |  | 5.62 [3.16 - 10.0] |  |
| Epithelioid sarcoma (8804/3) | 85 | 26 | 2.16 [1.40 - 3.34] |  | 2.59 [1.66 - 4.05] |  |
| Leiomyosarcoma (Excluding Skin) (8890/3) | 48 | 2 | 0.24 [0.06 - 0.98] |  | 0.30 [0.07 - 1.22] |  |
| Liposarcoma, NOS (8850/3) | 50 | 7 | 0.91 [0.42 - 1.97] |  | 1.82 [0.81 - 4.05] |  |
| Malignant peripheral nerve sheath tumor (9540/3) | 229 | 86 | 2.78 [2.07 - 3.73] |  | 2.00 [1.46 - 2.73] |  |
| Other tumors | 188 | 38 | 1.39 [0.95 - 2.03] |  | 1.27 [0.86 - 1.88] |  |
| Undifferentiated sarcoma NOS (8805/3) | 227 | 61 | 1.87 [1.35 - 2.58] |  | 1.07 [0.74 - 1.54] |  |
| **Histological grade (FNCLCC)** |  |  |  | **<0.0001** |  | **<0.0001** |
| Grade 1 | 122 | 5 | — |  | — |  |
| Grade 2 | 375 | 68 | 4.42 [1.78 - 11.0] |  | 4.90 [1.90 - 12.6] |  |
| Grade 3 | 307 | 84 | 7.46 [3.03 - 18.4] |  | 4.86 [1.89 - 12.5] |  |
| Grade GX | 732 | 181 | 6.48 [2.66 - 15.8] |  | 5.17 [2.03 - 13.1] |  |

**Abbreviations:** *FNCLCC, Fédération Nationale des Centres de Lutte Contre le Cancer (French National Centers Federation against cancer); NA, not available; NOS, non-other specified; SC, spindle cells, T1, Tumor confined to tissue of origin; T2, Tumor extending beyond tissue of origin; Tx, Tumor extent unknown.*

**Supplemental Table 2.** Risk factors associated to overall survival for localized tumors (univariate and multivariate analyzes).

Fig 1a Fig 1b


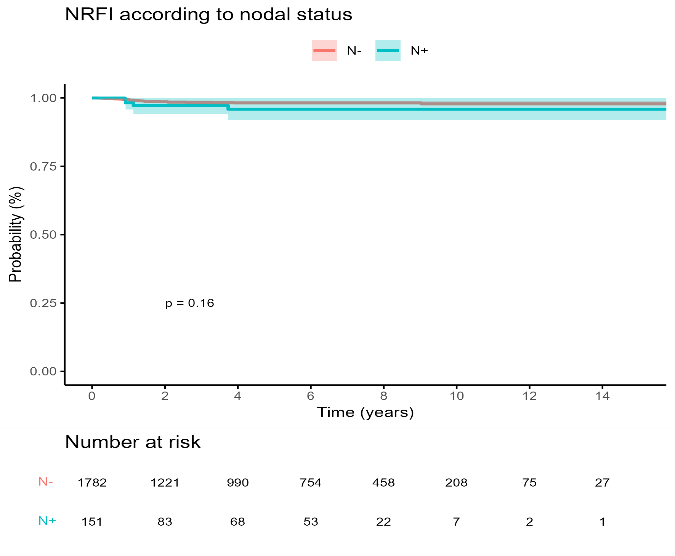

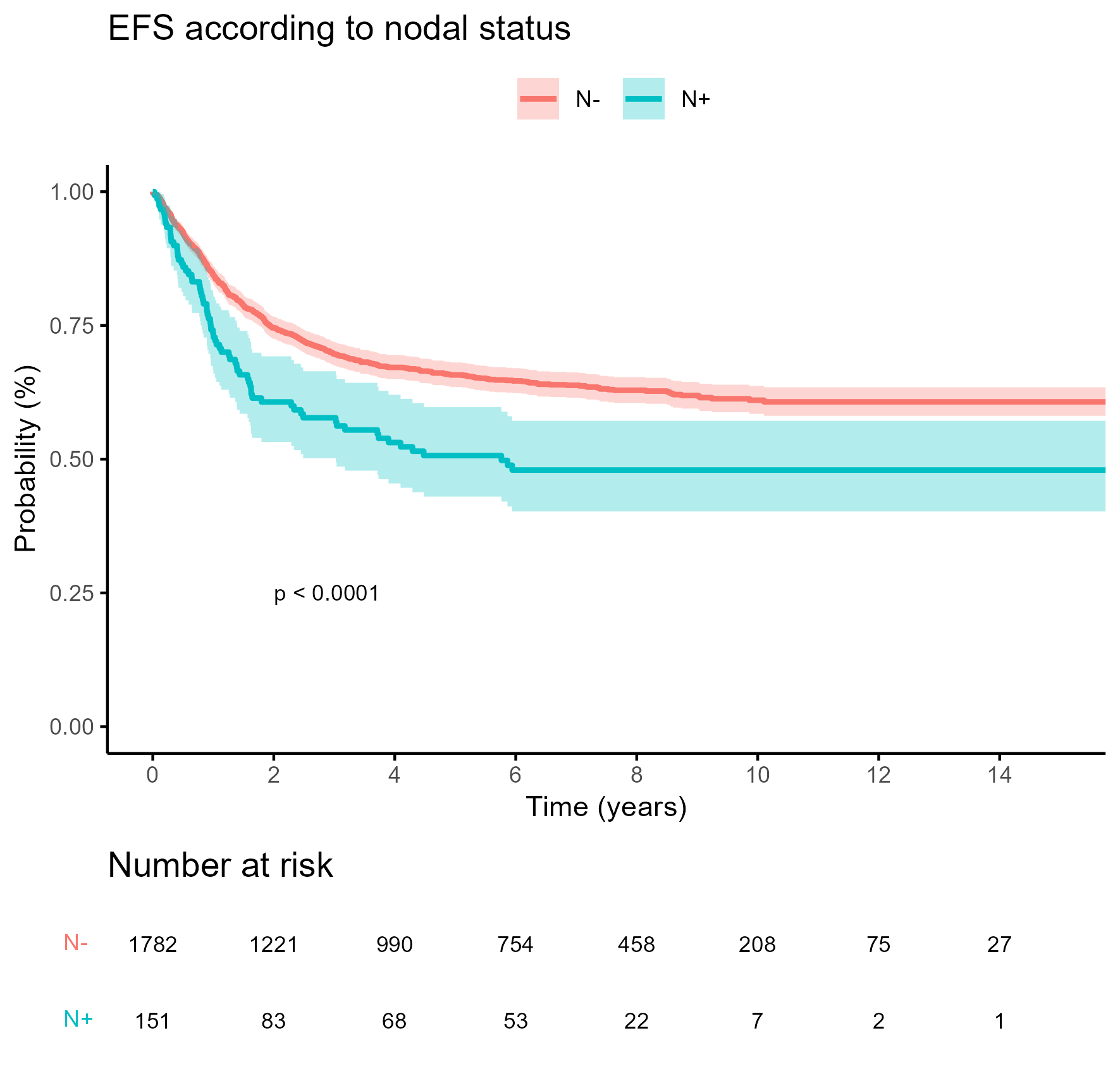


Fig 1c Fig 1d


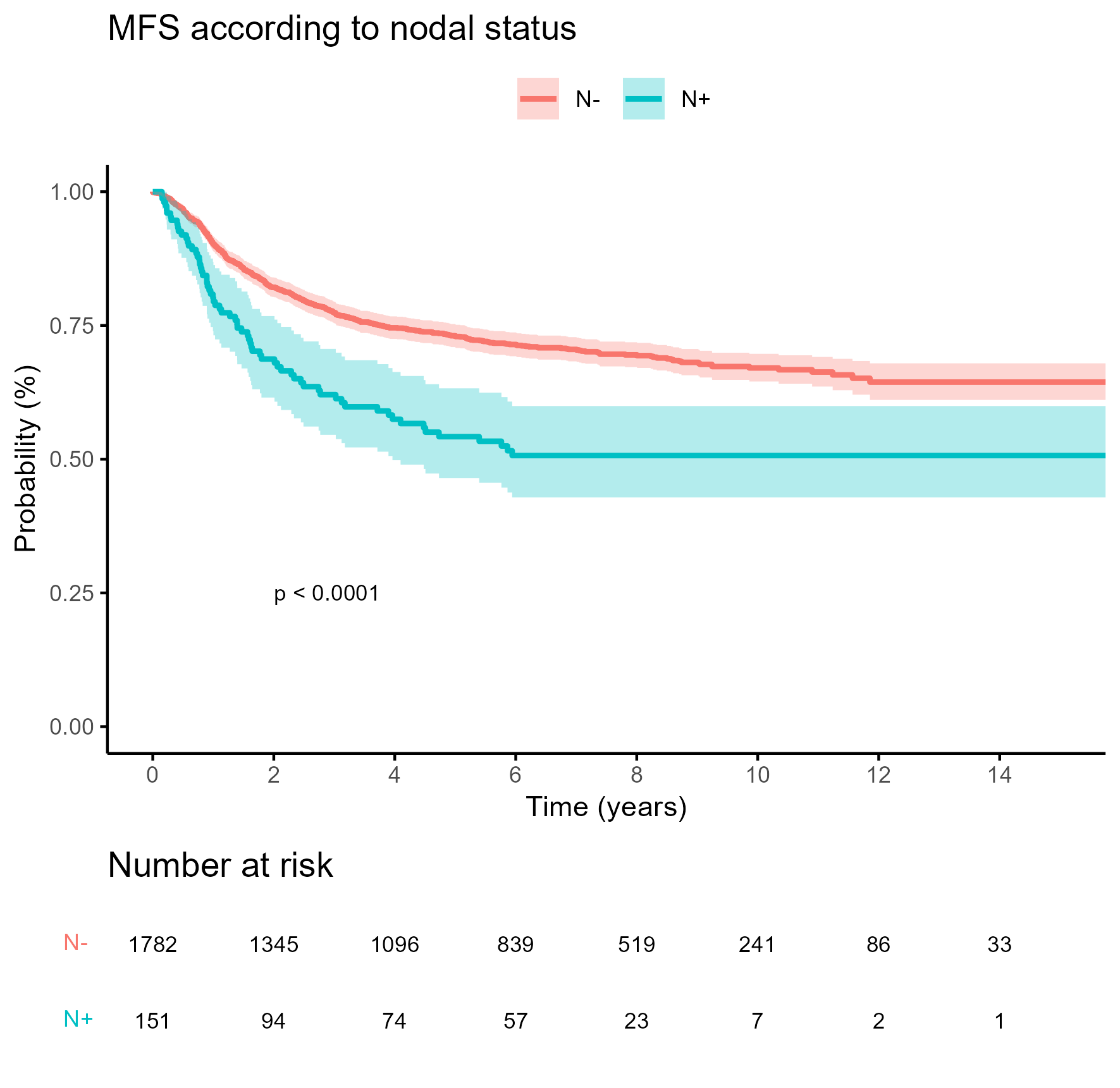

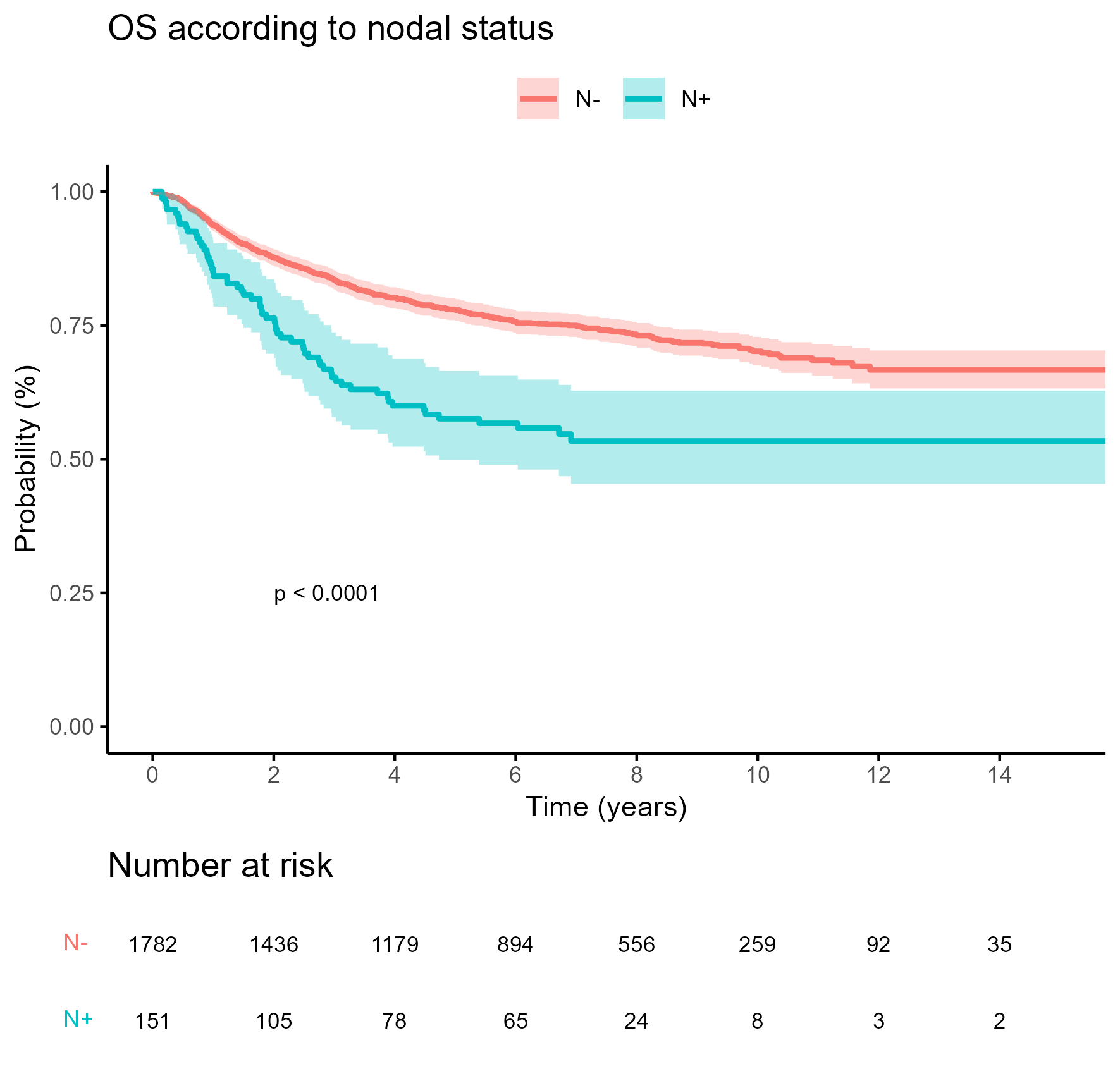


*Red line: no N1 at diagnosis, blue line: Presence of N1.*

**Supplemental Figure 1.** Impact of the regional lymph node invasion (N1) for the entire cohort of NRSTS. *Nodal relapse free interval (fig 1a), Event free survival (fig 1b), Metastasis free survival (fig 1c) and Overall survival (fig 1d).*
